# Analysis and automatic detection of lava flows using SAR backscatter applied to the 2017 eruption of Erta ‘Ale Volcano, Ethiopia

**DOI:** 10.1007/s00445-026-01984-8

**Published:** 2026-05-08

**Authors:** Jemima Gosling, Edna Warsame Dualeh, Juliet Biggs

**Affiliations:** COMET, School of Earth Sciences, University of Bristol, Bristol, BS8 1RJ UK

**Keywords:** Lava flow mapping, Synthetic Aperture Radar (SAR) backscatter, COSMO-SkyMed, Automated detection, Erta ‘Ale, Ethiopia

## Abstract

Mapping lava flows from a range of conditions and environments is critical for understanding flow behaviour and hazard assessment, but ground-based observations can be challenging, especially in remote regions. Here, we use 39 SAR backscatter images from the COSMO-SkyMed satellite to measure the extent and surface properties of lava flows from the 2017 to 2019 eruption of Erta ‘Ale volcano, Ethiopia. Using pairs of SAR backscatter images, we produce change difference images and map the flow outlines. We observe both increases and decreases in backscatter, which we attribute to differences in surface roughness. These manual-derived flow maps produced detailed outlines that are consistent with results obtained from other datasets, such as coherence. We then apply a sequential analysis technique, CUSUM, to corrected backscatter timeseries and find that it can automatically identify flows with an accuracy of 88%. Automated detection provides an objective and repeatable alternative to manual mapping. We find that at least four pre-eruption acquisitions are required as a baseline to achieve a robust classification. Our results demonstrate that SAR backscatter timeseries analysis can be used to characterise lava flow emplacement and surface evolution in remote volcanic environments.

**Supplementary Information:**

The online version contains supplementary material available at 10.1007/s00445-026-01984-8.

## Introduction

Lava flows are a common feature of volcanic eruptions, and whilst they cause fewer fatalities than other, faster travelling volcanic flows (e.g., pyroclastic density currents), they still have the potential to threaten infrastructure and populations (e.g., Harris 2015; Hulme 1974; Kilburn 2015; Walker 1973). Observing lava flows aids in developing an understanding of their behaviour, helping to determine warning signs and the rates and extent of potential hazards. Accurate and timely lava flow maps are critical resources for use in volcanic monitoring. In particular, they can be used as test datasets to validate and develop lava flow models, which have both short- and long-term applications such as eruption forecasting (e.g., Hyman et al. 2024), hazard assessment (e.g., Ezquerro et al. 2023), and land use planning (e.g., Chevrel et al. 2021). However, accurate mapping and monitoring require consistent, widespread observations, which are highly time-consuming and expensive to collect using ground-based techniques. Only 50% of active volcanoes have ground-based monitoring instruments (Brown et al. 2015), and many volcanoes are located in remote regions with rough terrain, meaning observations can be insufficient or non-existent. Further, field surveys can be dangerous during eruptions; therefore, observations of lava flows are often constrained to the final flow, and information on their progression and behaviour is lost.

Space-borne remote sensing provides rapid, efficient, high-resolution observations of flow emplacement during and after eruptions. Remote sensing of lava flows is done primarily using high-resolution optical imagery (e.g., Chevrel et al. 2023; Gouhier et al. 2022), thermal (e.g., Blackett 2017; Coppola et al. 2015; Wright et al. 2004), and Synthetic Aperture Radar (SAR) instruments (e.g., Ebmeier et al. 2018; Lee et al. 2023; Woodhouse 2017). Moderate resolution thermal imagery can observe thermal anomalies and hotspots that can indicate volcanic activity and can be used to derive a time-averaged discharge rate (TADR) (Coppola et al. 2013) and estimate erupted volumes (Coppola et al. 2025). However, these estimates may underestimate the actual TADR in the presence of ash or cloud cover, which are common during eruptive events.

**Fig. 1:**
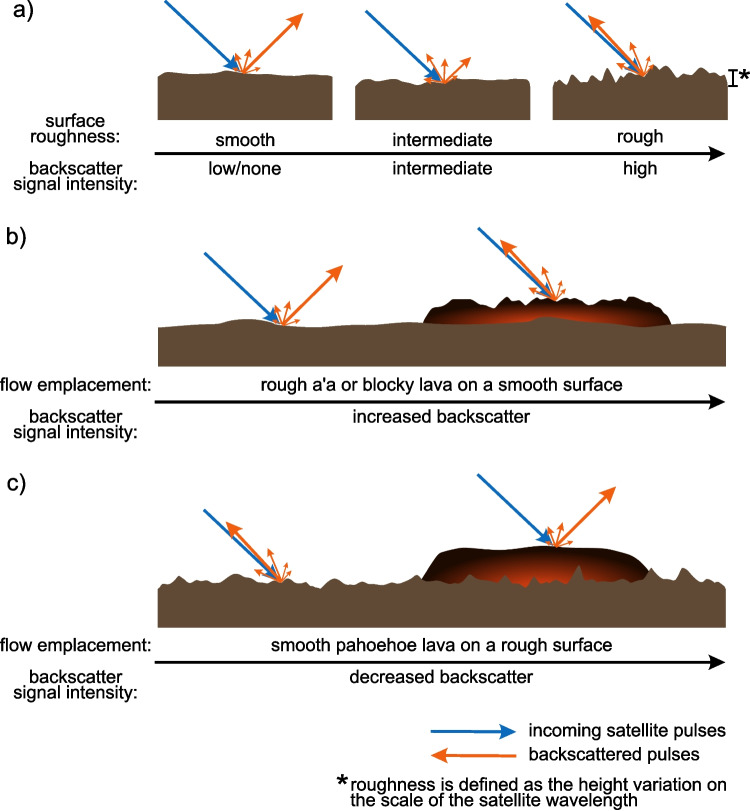
Schematic showing how SAR backscatter is affected by changing surface roughness with arrow length representing the relative backscatter energy: **a**) increasing surface roughness intensifies backscatter; **b**) emplacement of a rougher textured lava flow than the surface beneath it will increase backscatter; **c**) emplacement of a smoother textured lava flow than the surface beneath it will decrease backscatter. Note: the local topography and dielectric properties of the surface also affect backscatter signals, but their effects are generally negligible for lava flow deposits

Lava flow emplacement changes the surface topography and characteristics, altering both the amplitude and phase components of SAR images. Interferometric SAR (InSAR) coherence images can be used for flow mapping (e.g., Dietterich et al. 2012; Moore et al. 2019; Polcari et al. 2025), but only where baseline coherence is good. This depends on the temporal sampling and wavelength of the SAR dataset (e.g., Dietterich et al. 2012; Zebker et al. 1992). However, if flows interact with incoherent areas (e.g., highly vegetated or snow-covered; Ebmeier et al. 2018), the use of coherence becomes more complex.

SAR backscatter imagery has the potential to provide details of the surface characteristics of the flow, as it is sensitive to surface roughness (Pinel et al. 2014) and can provide information where optical and thermal imagery are limited by cloud coverage and InSAR coherence is poor. However, InSAR methods are now widely used in volcanology, whereas SAR backscatter methods and systematic analysis are less developed, mainly due to uncertainties in interpretation. The increasing number of high-resolution X-band sensors, e.g., COSMO-SkyMed (CSK), ICEYE, TerraSAR-X, and improved access through organisations such as the Committee on Earth Observation Satellites (CEOS) and the GEO Geohazard Supersite and Natural Laboratory initiative (GSNL) (e.g., Pritchard et al. 2018a) mean the development of SAR backscatter analysis for lava flow mapping is now timely (Zhang et al. 2019).

SAR backscatter changes in response to surface roughness, moisture, and topography (Pinel et al. 2014). For volcanic flows, the dominant cause of SAR backscatter signals is changes to the surface roughness. The influence of surface roughness on SAR backscatter depends on its scale relative to the radar wavelength. Features smaller than the wavelength tend to appear smooth, scattering energy away from the SAR sensor, while surfaces comparable to the wavelength scatter more energy, returning more to the sensor and producing a higher backscatter signal (Fig. 1). Large-scale roughness or slope variations also influence the observed signal; however, when these remain relatively constant or vary over larger areas, wavelength-scale roughness remains the dominant factor. Sensor wavelength is therefore an additional consideration, as it determines the scale at which surface roughness is reflected in SAR imagery. Therefore, we expect that different types of flows would produce different signals that could be used to identify various deposit types such as ash, lava, and pyroclastic flows (Dualeh et al. 2021). For example, we expect smooth pahoehoe flows to have lower backscatter signals than rough ‘a‘ā flows (Fig. 1b, c). Both increases and decreases in backscatter have been observed in lava flows, attributed to changes in roughness, including surface smoothing by ash deposits (Goitom et al. 2015), or changes in slope (Dualeh 2022). SAR backscatter images have previously been used individually to map lava flow emplacement and thicknesses (Dumont et al. 2018; Wadge et al. 2011) or compared to show changes through time indicating flow deposits (Goitom et al. 2015; Plank et al. 2019) and changing effusion rates (Arnold et al. 2017; Wadge et al. 2012). Importantly, they have been used to inform life-saving decisions during eruptions (e.g., dome growth and collapse, Pallister et al. 2013). Furthermore, combining SAR backscatter with coherence products to generate SAR Volcanic Flow Maps (VFMs) can capitalize on the strengths and compensate for the weaknesses of individual data types (Poland 2022).

Here, we analyse 39 SAR backscatter images from the COSMO-SkyMed (CSK) satellite covering the 2017–2019 eruption of Erta ‘Ale, Ethiopia, which produced $\sim$ 26 km$^2$ of basaltic flows (Moore et al. 2019). Erta ‘Ale’s arid environment represents a contrasting environment from the lava flows previously studied with SAR backscatter, which have primarily been in tropical environments where weathering and vegetation growth can cause rapid changes to backscatter (e.g., Arnold et al. 2017; Dietterich et al. 2012; Wadge et al. 2012). This allows for systematic analysis of SAR methods without external complexity. We compare mapping using manual image inspection with an automated cumulative sum (CUSUM) sequential analysis technique (Page 1954) and discuss the results and effectiveness of each method. Finally, we investigate lava flow morphology changes across the flow field and the origin of the changes in backscatter.

## Dataset and methods

### Case study eruption: Erta ‘Ale, Ethiopia

**Fig. 2:**
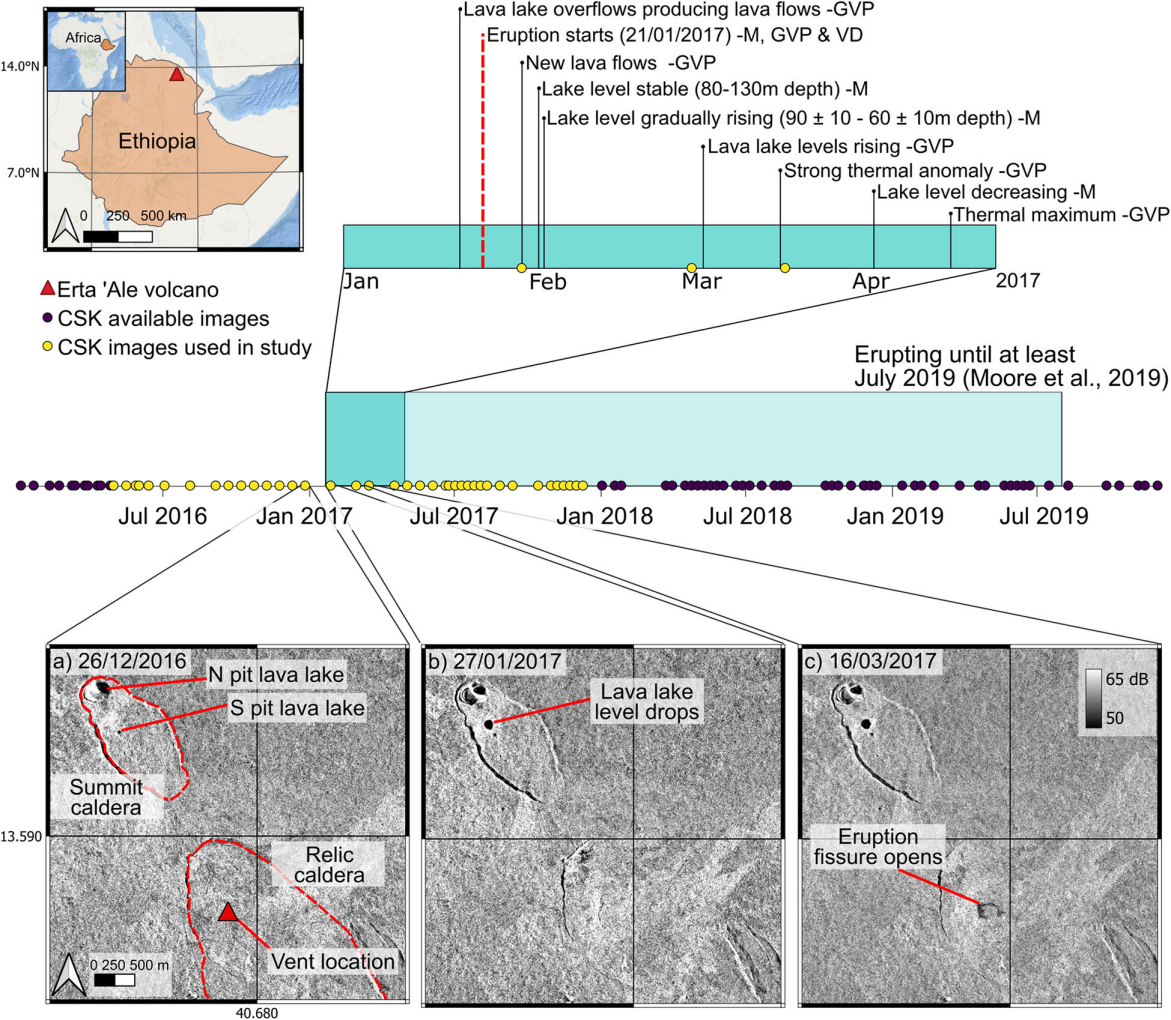
Case study overview: Erta ‘Ale location and timeline of the 2017 eruption showing early eruptive events identified from the literature from Moore et al., 2019 (M), Global Volcanism Program (GVP), and Volcano Discovery (VD), plotted alongside COSMO-SkyMed (CSK) image availability (circles) highlighting those analysed in this paper (yellow circles). **a**–**c**) Georeferenced SAR backscatter images showing **a**) main features of Erta ‘Ale; **b**–**c**) early eruptive events visible in images

Erta ‘Ale lies at the northern end of the East African Rift System (EARS) in the Afar region of Ethiopia (Fig. 2) (Biggs et al. 2021). The Erta ‘Ale spreading segment forms a 120-km-long ridge separating the Danakil microplate from the Nubian plate and is made up of distinct volcanic centres, including Erta ‘Ale and Alu Dalafilla, where shallow axial magma chambers feed basaltic pahoehoe style lava flows (Pagli et al. 2012). Erta ‘Ale has been in its current eruptive period since 1967, exhibiting lava flows in November 2010, between January 2017 and July 2019 (Field et al. 2012; Moore et al. 2019) and July 2025, the most recent eruption (La Rosa et al. 2025). Erta ‘Ale has no continuous ground-based monitoring, and most observations are either from field surveys (e.g., Global Volcanism Program 2017) or satellite observations (e.g., Field et al. 2012; La Rosa et al. 2025; Moore et al. 2019; Xu et al. 2017). There are two persistent lava lakes (north and south pits; Fig. 2a) which offer insight into the magma chamber, through thermal (Vergniolle and Bouche 2016) and depth fluctuations, related to pressure within the system (Moore et al. 2019). The south pit lake exhibited large fluctuations in January 2017, culminating in its overflow shortly before eruptions began from a newly formed fissure vent to the SE (Global Volcanism Program 2017) on 21 January (Fig. 2). Sentinel-1 SAR imagery from the 2017 eruption has been used to investigate the shallow axial plumbing system (Moore et al. 2019; Xu et al. 2017), coherence images to map flow progression, and radar shadows to estimate lava lake levels (Moore et al. 2019). InSAR coherence maps show that the eruption continued until at least July 2019 and saw lava flows extending to the NE and SW with an estimated total flow coverage of 26 ± 1 km$^2$ (Moore et al. 2019). Analysis of the TanDEM-X Digital Change Map gave an average thickness of 6.9 m, with the greatest height changes around the lava lake (44.2 ± 0.2 m) and in the north-east where the lava flows pool in a region of flatter topography (27.3 ± 0.2 m) (Edwards and Biggs 2025). This corresponds to a total volume of $173 \pm 6 \times 10^6$ m$^3$ across an area of 25.3 km$^3$, which is an order of magnitude higher than that estimated by Moore et al. (2019) who assumed an average flow thickness is between 0.5 and 2.5 m across the entire flow area based on field reports.

### SAR dataset and pre-processing

COSMO-SkyMed (CSK) SAR data was provided through the European Space Agency’s (ESA) third-party mission (TPM) scheme and CEOS Volcano Demonstrator (Pritchard et al. 2018b). The CSK constellation consists of four X-band satellites operating at a wavelength of 3.1 cm. Data was acquired on a right-looking, ascending track in stripmap mode and horizontal-horizontal (HH) polarisation. We processed 39 images (HI_03) spanning between April 2016 and December 2017 (Fig. 2), covering the first year of the eruption. Using GAMMA software (Werner et al. 2000), we produced backscatter geotifs from amplitude components of the raw SAR data, resampled to a target area spanning the lava flows and multi-looked (4$\times$4 pixels; resolution: 4$\times$9 m, Fig. S1) to reduce noise, such as speckle. To remove any geometric effects caused by the topography, we applied a radiometric terrain correction (Small 2011).

We used an oversampled 30 m resolution DEM generated by NASA’s Shuttle Radar Topography Mission (SRTM) (Farr and Kobrick 2000) for multi-looking and geocoding (Fig. S1). The STRM DEM has a coarser resolution than the SAR imagery being analysed; however, due to the lack of significant topography changes in the region, this did not significantly affect our analysis. At Erta Ale, between 2000 and 2017, the overall topography has remained relatively unchanged, with changes largely confined to the summit caldera. However, in more dynamic environments where the topography has changed (e.g., from lava flow emplacement, dome growth and collapse, and slope failure), using an outdated DEM could distort the results. In such cases, it would be preferable to use a newer DEM or to omit the terrain correction, especially if areas of change potentially overlap with the process being examined. Similarly, geocoding the images is not essential for the automatic change detection, and the methods applied here work equally well on images in radar coordinates. We applied the methods to geocode the images for convenience and interpretability. Given the low relief and arid environment at Erta ‘Ale, the terrain correction and DEM resolution and acquisition have minimal impact on our results (Fig. S1).

We observe a common signal across the whole image, even in areas that should remain unchanged through time. This common signal exists across all data points and could be attributed to seasonality, data processing, and/or noise. In our dataset, this pattern is likely related to differences between individual CSK satellites (Dualeh 2022). When this signal dominates, it may mask smaller variations. Therefore, to account for any common or widespread signal, we perform Principal Component Analysis (PCA) to identify and mitigate for these noise sources. Before applying PCA, we use boxcar averaging (resolution  20$\times$20 m) to reduce speckle noise while enhancing common signals (Fig. S1). Speckle is formed from coherent interference of the backscattered signal, producing a grain-like noise texture that can obscure slower-varying signals. There are various filters and algorithms for despeckling from simple averaging (e.g., multi-looking) to spatial filtering (e.g., updated Lee Sigma filter, Lee et al. 2008) to deep-learning approaches (e.g., Valade et al. 2023). We apply a simple boxcar filter as it smooths out rapid fluctuations while preserving larger-scale spatial features (Fig. S1). By applying this pre-processing step, we ensure that the PCA extracts meaningful variations rather than speckle-related patterns. We apply PCA on georeferenced images rather than in radar geometry and then geocode. At Erta ‘Ale, we did not observe a noticeable difference in PCA performance depending on input. However, further testing would be needed for case studies with more prominent topography, as geocoding may introduce distortions that incorporate artifacts identified during PCA. After averaging, we remove the first principal component from the dataset, which here accounted for 80% of the variation in the dataset (up from 51% without boxcar averaging).

### Manual image analysis

Backscatter variations may be visible in individual images, but in many cases, the signal needs to be augmented, for example, by analysing changes between images to remove background variations. We performed a logarithmic transformation to convert the backscatter images into decibels (dB). This accounts for the high dynamic range of the data. To analyse changes in signal, we produce ratio, $$r = \mid \left( \frac{i_{1}}{i_{2}}\right) \mid $$, and difference, $$ d = i_{2} - i_{1}$$, images between consecutive pairs, where $$i_{1}$$ is the earlier image in the time series and $$i_{2}$$ is the more recent acquisition. To further visualise these changes, we produced RGB (red, green, blue) change difference images, based on the method described in Wadge et al. (2011), placing images in separate bands: R = $$i_{2}$$, G = $$i_{1}$$, B = ratio (*r*). RGB images highlight areas where backscatter increases (red), decreases (blue), or remains unchanged (yellow). Similar to the application of PCA, we form the geotiffs directly from the georeferenced image, as the gentle topography at Erta ‘Ale means that the order of these steps did not produce a noticeable difference in outputs. Finally, we calculated the variance of each pixel across the full time series, as we expect greater variance within flow boundaries compared to the background areas. We use all these images together to manually plot flow boundaries in QGIS, assuming flows are defined by where backscatter changes between images, producing a series of shapefiles of the flow progression. We also calculate the local topographic slope across the mapped flows to evaluate the influence of changing topography on lava flow morphologies and backscatter signals.

### Time series analysis

Flow emplacement causes deviations of backscatter amplitude from the background values. We determine the date of flow emplacement on the assumption that there will be a step change in backscatter at this time. However, background noise may also cause deviations of the backscatter amplitudes through time, so a single deviation may not imply flow emplacement and lead to false flow detection. First, we visually inspect the time series formed from individual SAR images. We then apply an automatic detection technique.

We applied the cumulative sum (CUSUM) algorithm (Page 1954) to our PCA-corrected backscatter timeseries to detect small deviations from a specified background level, or baseline. CUSUM is a sequential change-detection method that identifies small but sustained shifts in the mean of a timeseries by accumulating deviations from a target mean. It has previously been applied to InSAR timeseries to automatically detect deformation (Albino et al. 2020). For each individual pixel, we calculate the mean ($\mu$) and standard deviation ($\sigma$) using a time series of pre-eruption backscatter values. We refer to this as our baseline period. These baseline statistics were used as inputs for MATLAB’s +cusum+ function (Signal Processing Toolbox), which internally normalises the timeseries with respect to the baseline mean and standard deviation, similar to normalising the timeseries $z_i$=($x_i$-$\mu$)/$\sigma$, where $x_i$ is the backscatter value at time step *i*. We apply a two-sided CUSUM test, which calculates two cumulative sums (1) for positive shifts ($I_i$) and (2) for negative shifts ($D_i$)

1 $$\begin{aligned} I_{i} = {\left\{ \begin{array}{ll} 0 & i = 0 \\ \text {max}(0, I_{i-1} + z_{i} - 0.5k) & i > 0 \end{array}\right. } \end{aligned}$$

2 $$\begin{aligned} D_{i} = {\left\{ \begin{array}{ll} 0 & i = 0 \\ \text {min}(0, D_{i-1} + z_{i} - 0.5k) & i > 0 \end{array}\right. } \end{aligned}$$

where k represents the minimum shift, in standard deviations, that the algorithm treats as significant. The detection threshold is applied after computing the cumulative sums, and a change is flagged when either $I_i$ or $D_i$ exceeds this control limit. Based on preliminary testing across representative individual pixel timeseries, we set the sensitivity shift (k=2$\sigma$) and detection threshold (5$\sigma$). These settings were found to detect meaningful changes while minimising false detection driven by short-term variability and noise. Further testing would be required to determine threshold values that are transferable across different eruptions, sensors, and environmental settings.

At Erta ‘Ale, we first applied CUSUM to a subset of pixels, previously analysed during the manual inspection, and then extended the analysis to all pixels within the study area to automatically identify deviations and the associated date of each detected change. We use the pre-eruption images as input for calculating the baseline statistics.

## Results

### Manual image analysis

Individual backscatter images clearly show the early eruptive activity, which includes a drop in the lava lake levels and the formation of an eruption fissure (Fig. 2a–c), as documented by Moore et al. (2019). These events caused significant changes to the land surface and decreases in backscatter values, making them clear during individual image inspection. In the individual CSK images, the drop in the lake levels and a fissure on the relic caldera are visible in the first post-eruption acquisition (27-01-2017). This coincides with previous remote sensing observations (thermal, gas, and deformation), that the relic caldera fissures opened between 19 and 21 January 2017 (Global Volcanism Program 2017; Xu et al. 2017). Lava flow emplacement causes visible change between consecutive images, but the low magnitude variation between backscatter signals of the flows and background means individual images cannot be used to accurately map flow progression.

**Fig. 3:**
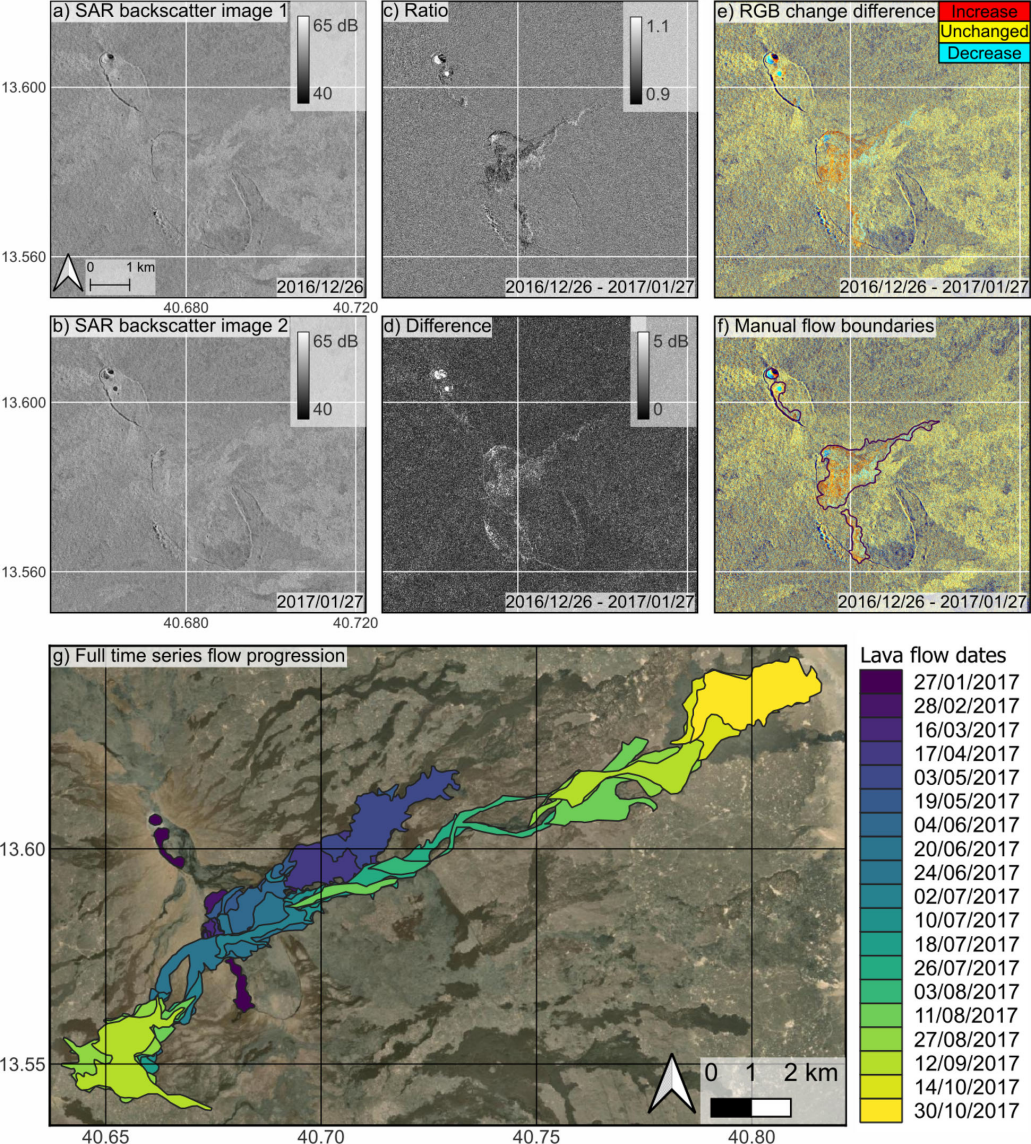
Backscatter images (**a**–**b**), image analysis methods (**c**–**f**), and manual flow map (**g**): Terrain corrected COSMO-SkyMed SAR backscatter images of Erta ‘Ale (**a**) pre- and (**b**) post- eruption start; **c**) Absolute ratio and (**d**) difference between acquisitions (**a**–**b**) to emphasise areas of backscatter change; **e**) RGB change difference image of these acquisitions and their ratio. Backscatter increases appear red, decreases appear blue, and unchanged areas appear yellow, highlighting areas of active flows; **f**) example manual flow boundary identified through visual inspection of RGB images; **g**) full manual flow map showing the progression of lava flows from January to October in the 2017 eruption of Erta ‘Ale. RGB manual flow outlines were produced individually and combined for full flow boundaries. Note: flow boundaries were unclear in RGB images after October 2017 and so were excluded from manual mapping

Ratio (Fig. 3c) and difference (Fig. 3d) images emphasise backscatter change, highlighting flow emplacement; however, background noise makes flow boundaries hard to define accurately using ratio or difference images. RGB change difference images (Fig. 3e) help to distinguish new flow regions and visually describe how the backscatter changes between images, with increases and decreases observed between most image pairs (Fig. 3f, g). At Erta ‘Ale, backscatter was more uniform across the flows than the background, and time series variance was greater within the flows than background pixels (1.0 ± 3.0 dB and 0.6 ± 5.7 dB, respectively) and could be used to map the final flow outline (Fig. 5a). However, channelised flow regions less than 300 m wide were difficult to map using RGB images alone. This causes slight differences between flow regions highlighted in variance images and those identified from RGB images. The analysis also shows the highest variance at the furthest ends of the flows to both directions (Fig. 5a).

The final flow maps (Fig. 3g) show flow emplacement progressing to the NE and SW directions as previously reported (Moore et al. 2019), with flow emplacement to the NE from the beginning of the eruption until at least October 2017 and additional emplacement towards the SW following the 04-06-2017 acquisition (Fig. S2). In total, the flows extended 17 km to the NE and 5.7 km to the SW, covering a total area of 27.5 km$^2$. These estimates agree with previous InSAR measurements of 26 ± 1 km$^2$ for flows emplaced between January 2017 and July 2019 (Moore et al. 2019). However, the SAR backscatter maps cover a shorter period of the eruption, between January and October 2017, suggesting that either (1) InSAR coherence techniques underestimated flow coverage, (2) manual backscatter mapping overestimated coverage, or (3) little additional area was covered between October 2017 and July 2019. The final flow maps from these two methods are very comparable, with most difference located on the edges of the flow and where the lava was pooling. This suggests that most of the flow area had been covered by October 2017.

**Fig. 4:**
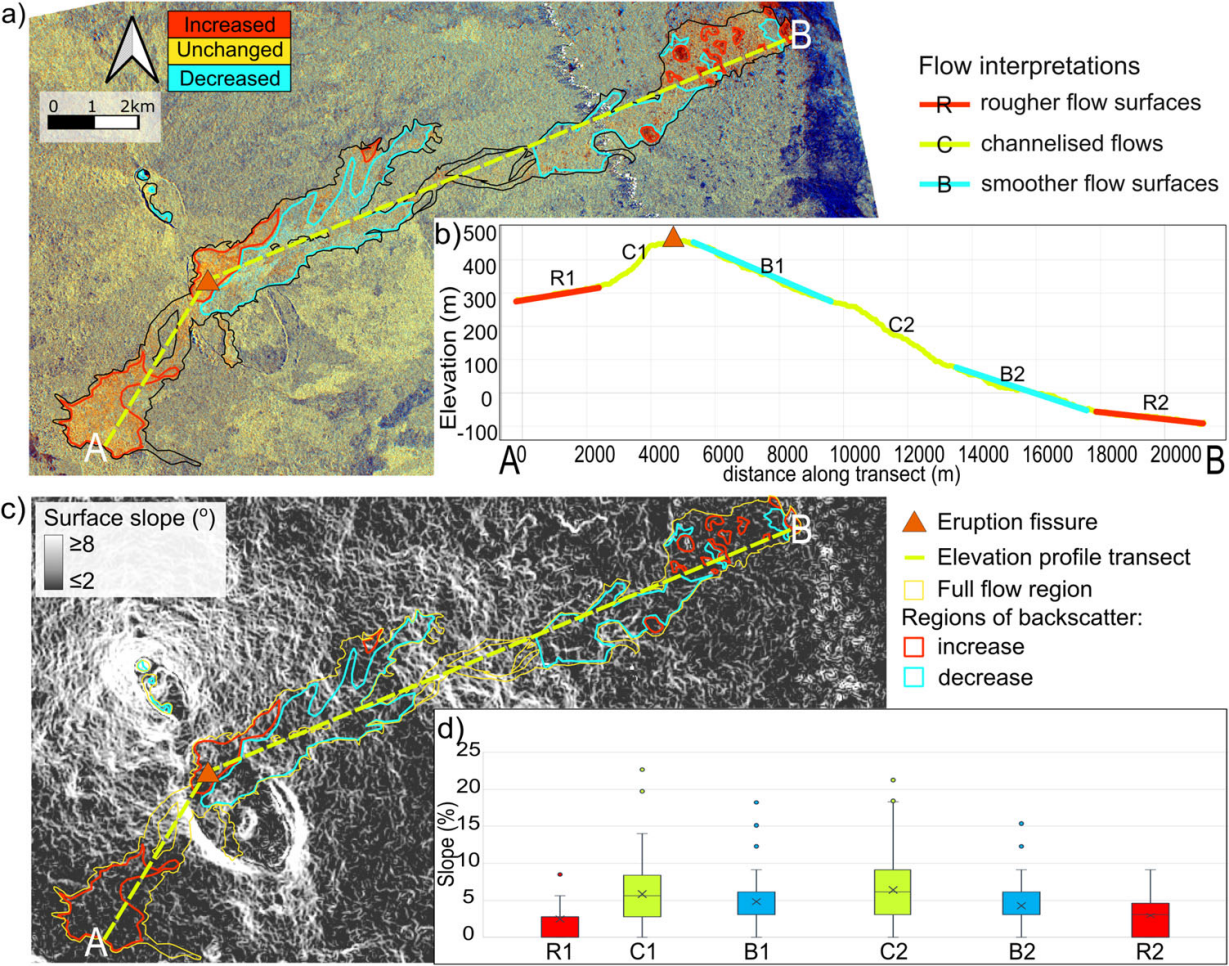
Backscatter variations with topography at Erta ‘Ale: **a**) RGB change difference image comparing first (30-04-2016) and last (09-12-2017) images, highlighting significant areas of backscatter increases (red) and decreases (blue); **b**) elevation profile along flow transect (lime dashed line) relating slope to backscatter variations; **c**) slope analysis of the flow area; **d**) box and whisker plots of slope within each region defined in **b**

Flow emplacement caused both increases and decreases in backscatter, with decreases mostly occurring close to the vent and increases at the ends of the flows (Fig. 4a). Slope analysis showed increases in backscatter were associated with the shallowest slopes (typically $\le$3$^{\circ }$), decreases in backscatter associated with intermediate slopes (typically 3-6$^{\circ }$) and in regions of steep slopes (typically exceeding 6$^{\circ }$) flows became very narrow and channelised and backscatter change was neither dominated by increases nor decreases (Fig. 4).

### Time series analysis

**Fig. 5:**
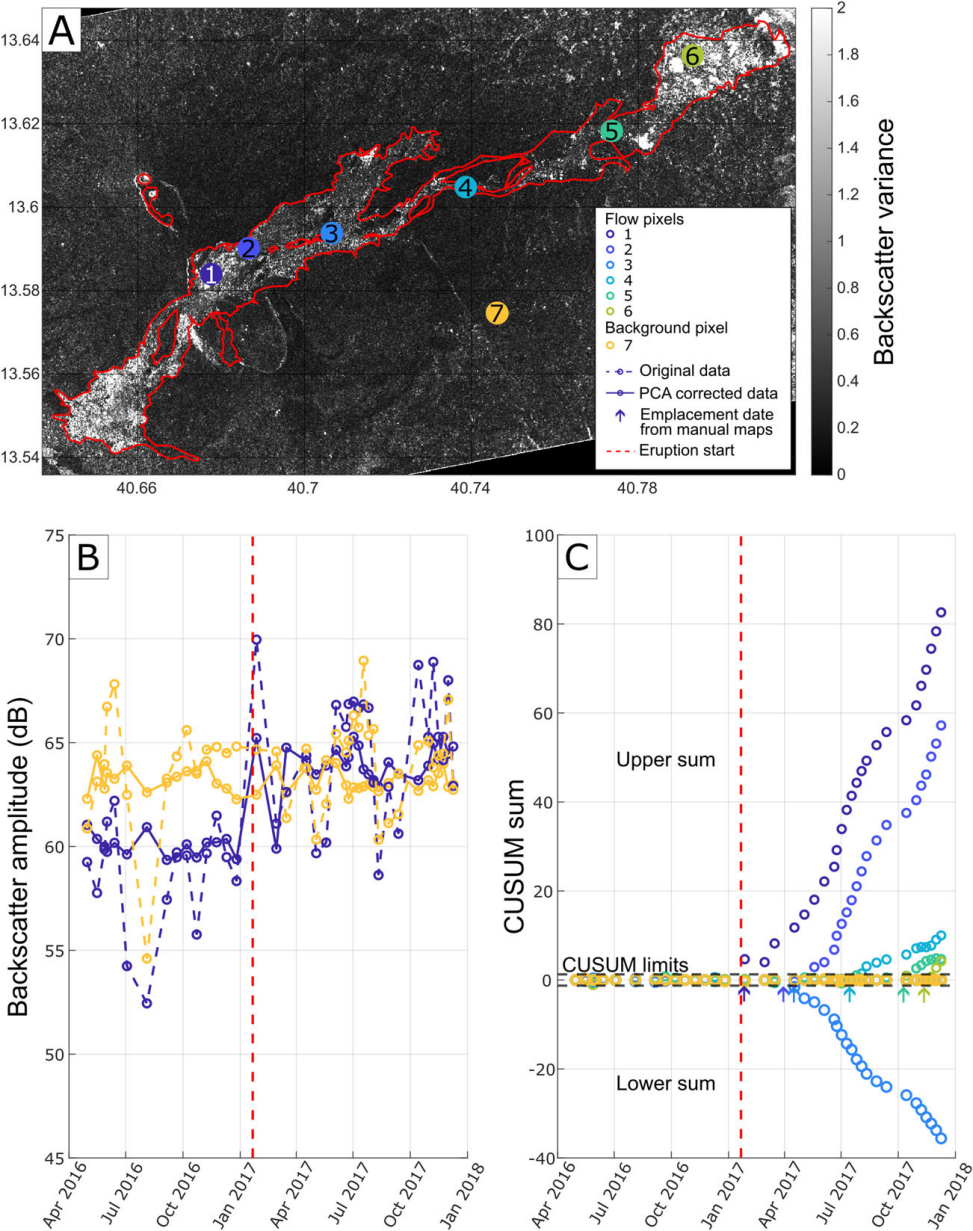
Comparison of flow emplacement detection manually and using CUSUM: **a**) variance image of amplitude across the full time series (30-04-2016–09-12-2017) showing example pixel locations for flow and background pixels; **b**) pixel backscatter time series showing original data (dashed lines) and data with PCA correction (solid lines); **c**) pixel CUSUM chart showing upper and lower cumulative sums of deviations above CUSUMs detection thresholds and a comparison of CUSUMs emplacement date estimations (when sum crosses limit) with manually mapped emplacement date estimations (arrows)

We illustrate our time series analysis using pixels selected from the manually mapped flow regions and variance images (Figs. 3g and 5a). For both flow and background pixels, we observe increases and decreases to backscatter values within the range of 50–70 dB (Fig. 5b). Following the beginning of the eruption, the backscatter within the flow appears to shift to increased values compared with background pixels; however, manual distinction is difficult (Fig. 5b). Using automated CUSUM analysis, the background pixels did not deviate above CUSUM detection limits, whereas pixels within manually mapped flow regions did (Fig. 5c). CUSUM is therefore capable of automatically distinguishing flows from the background and can be applied to both positive or negative backscatter deviations. The emplacement dates determined with CUSUM partially agree with those determined by manual mapping. Some pixels are detected in the acquisition following emplacement (e.g., Fig. 5c: pixels 1 and 3). However, many are detected a few acquisitions later (e.g., Fig. 5c: pixels 2, 4, 5, and 6), suggesting the backscatter deviations caused by flow emplacement are minimal and require continuous change that can cause a timelag in the CUSUM analysis.

**Fig. 6:**
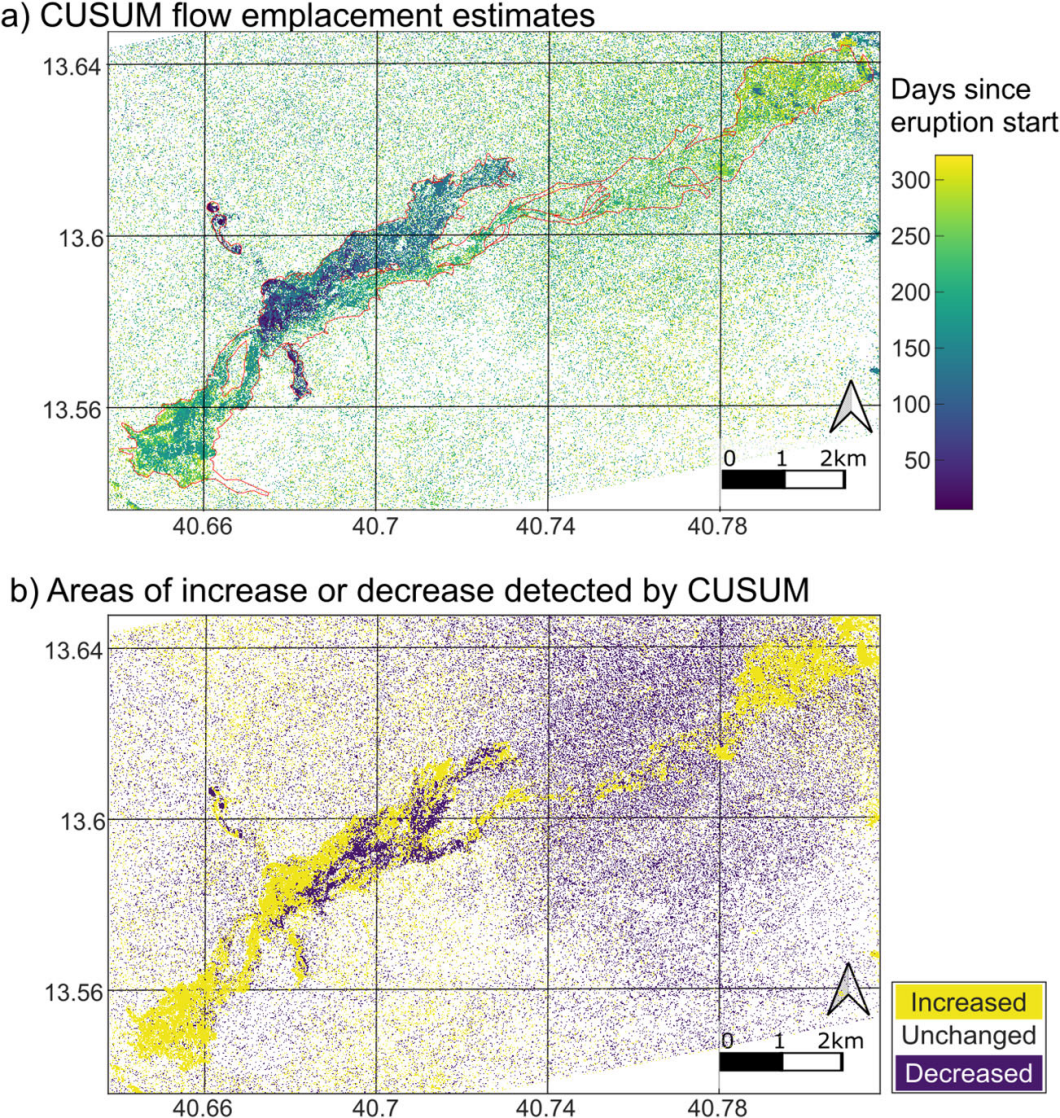
CUSUM results over full dataset area: **a**) CUSUM flow map with pixel colours corresponding to the time of flow emplacement estimated by CUSUM in days since the eruption start date (21-01-2017) compared to manually mapped full flow boundary (red outline); **b**) corresponding CUSUM map showing whether backscatter deviations exceeded upper (yellow) or lower (purple) CUSUM limits

When CUSUM is applied across the full image, we observed a gradient of flow emplacement dates from 0 to >300 days since the eruption start (purple to yellow) down flows to the NE and SW (Fig. 6a). Flow pixels mainly exceed the CUSUM upper threshold (increase in backscatter); however, in some regions, such as the lava lake and within the first 4 km of the NE flow, more pixels exceed the lower threshold (decrease in backscatter; Fig. 6b). CUSUM makes some false positive detections outside mapped flow boundaries, with twice as many of these having exceeded the lower threshold than the upper threshold. False negatives are also apparent, particularly in areas where flow channels are narrower (e.g., 13.61N, 40.74E).

**Fig. 7:**
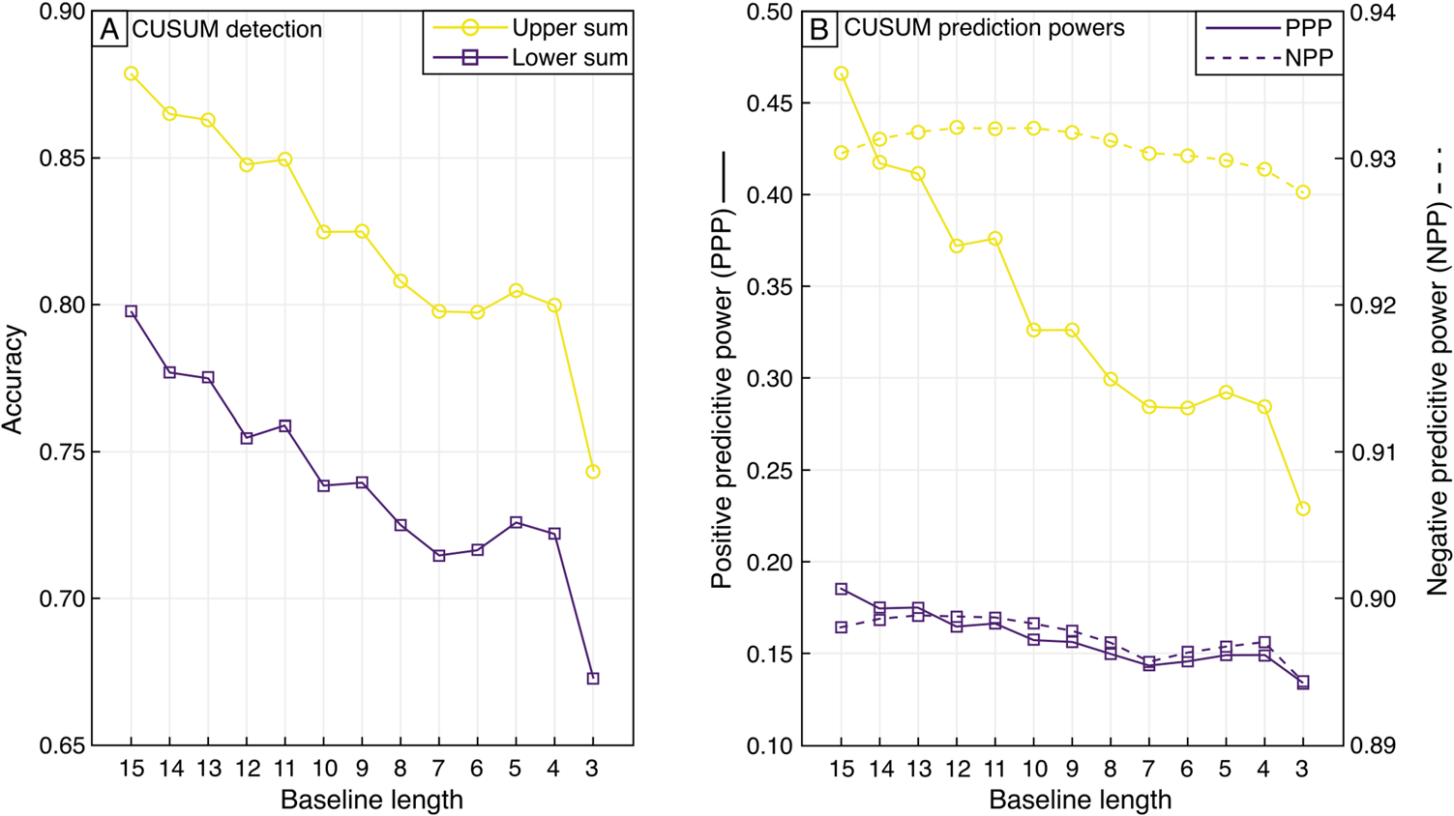
Effects of changing baseline length on CUSUM upper (yellow) and lower (purple) flow detection results: **a**) detection accuracy, i.e., the proportion of correctly classified pixels; **b**) positive (solid lines) and negative (dashed lines) predictive powers of CUSUM, i.e., the proportion of correctly detected flow (positive) and background (negative) pixels

We conducted a statistical analysis to evaluate CUSUM’s capabilities as a predictive model (Beguería 2006) using a confusion matrix where our manual mapping results are used to define the actual state, the CUSUM results represent the predicted state and each pixel is classified as true positive (TP), true negative (TN), false positive (FP), or false negative (FN) (Fig. 7). Using the full baseline of 15 images, the positive predictive power (TP/(TP+FP)) was 0.66 when both upper and lower limits are included and 0.47 for the upper limit only. The negative predictive power (TN/(TN+FN)) was 0.81 when both upper and lower limits are included, and 0.93 for upper limits only (Fig. 7). This equates to an accuracy ((TP+TN)/(TP+FP+TN+FN)) of 0.79 for both upper and lower limits, and 0.88 when only the upper limits are considered. The application of spatial filtering had the strongest effect on the CUSUM-derived flow maps. Stronger filters reduced scattered detection and produced more conservative flow maps (e.g., larger gaps within flow, low false negatives detection). The PCA correction primarily increased the detection sensitivity, capturing a greater proportion of the flow, but with an increased background classification. We test how the number of baseline images affects CUSUM’s performance and find that to achieve over 80% detection accuracy requires a baseline of at least four images (Fig. 7).

## Discussion

### Surface roughness

Our observations of the 2017 lava flows at Erta ‘Ale show both increases and decreases in SAR backscatter, with decreases occurring on steep slopes close to the vent and increases on shallow slopes at the ends of the flows. In general, we expect SAR backscatter signals to be lower for pahoehoe than ‘a‘ā flows due to the differences in surface roughness on the scale of the radar wavelength. However, the observations contradict this and illustrate the challenges in interpreting backscatter changes that result from a combination of scattering effects (Fig. 1).

**Fig. 8:**
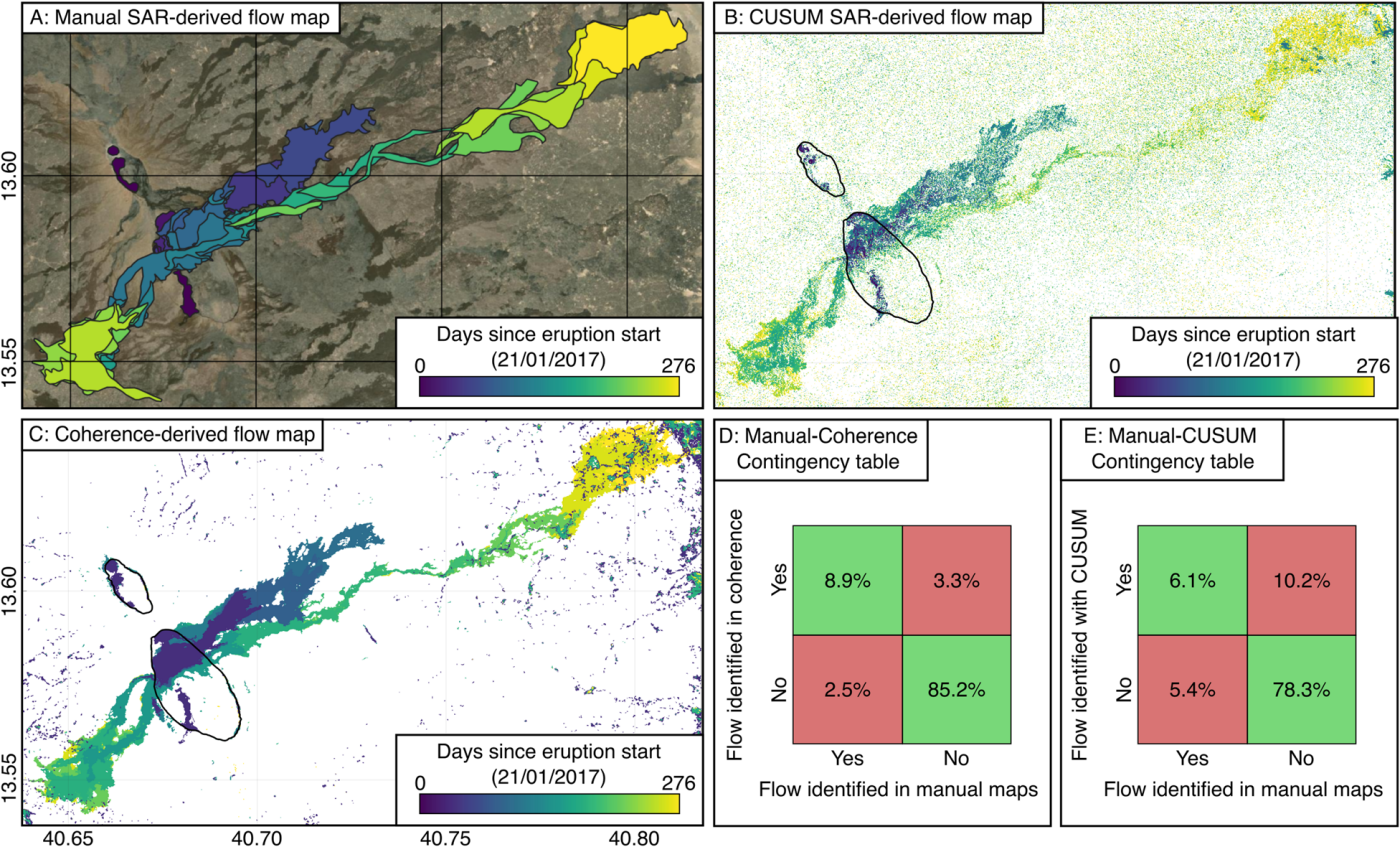
SAR backscatter and coherence-derived flow map comparison. Flow maps derived (**A**) manually from SAR images, (**B**) using CUSUM on backscatter timeseries, and (**C**) from coherence images. Statistical comparison shown as contingency tables between (**D**) manual- and coherence-derived maps and (**E**) manual- and CUSUM-derived maps

Satellite acquisitions only provide a snapshot in time of the flow surface, with inconsistent and sometimes long acquisition intervals meaning flow solidification or textural transitions can take place prior to the next satellite overpass (Fig. 2). Whether we see increases or decreases between images will also depend on the nature of the pre-existing surface. For example, pahoehoe lava flows from the 2011 eruption of Nabro volcano caused increases in backscatter because ash from the initial explosive eruption smoothed the surface, causing very low backscatter prior to the flow emplacement (Goitom et al. 2015). At Erta ‘Ale, the pre-existing surface was largely composed of old flows, and the eruption was purely effusive, which means changes in backscatter depend on the relative roughness of two generations of flows. Field photos suggest that although the flows are dominantly pahoehoe, rougher ‘a‘ā flows also occur in some areas. Together, these factors explain why we see both increases and decreases in surface roughness at Erta ‘Ale.

A key control on flow morphology is the topographic gradient. Topographic contributions to shear are lower on flatter slopes, and slope breaks disrupt lava flow surfaces, so rougher flow textures are expected on steeper slopes (Glaze et al. 2014). For example, the 2011–2013 flows from the Pu‘u ‘Ō‘ō crater, Kīlauea mainly decreased backscatter but increased backscatter was observed further from the vent and attributed to the fact that the lava flows had passed over an area of steep topography, increasing shear and causing transition to rougher ‘a‘ā textures (Dualeh 2022; Gregg 2017). We observe a similar phenomenon at Erta ‘Ale, where roughest flow textures are observed furthest from the vent on flatter slopes (Fig. 4a–c), signifying their solidification occurred after flowing down steeper slopes and over slope breaks (Fig. 4b). We also expect increased cooling with distance from the vent and therefore an increase in viscosity, which can also cause rougher morphologies and higher backscatter (Fig. 4).

### SAR Backscatter mapping techniques

#### Method comparison

Using manual inspection of CSK images, we produced high-resolution flow maps of the 2017 eruption at Erta ‘Ale (Fig. 3g). The maps are consistent with previously published maps produced using Sentinel-1 InSAR coherence analysis (Fig. S3, Moore et al. 2019), demonstrating that both methods capture the overall spatial extent of the lava flow emplacement. For a direct comparison, we generated flow maps from coherence images using the same CSK dataset used for the backscatter methods. This allows for an internally consistent comparison between mapping approaches. We found an overall good agreement between coherence-derived and the manual SAR-derived flow maps, with a Matthews correlation coefficient (MCC) of 0.7 and an accuracy of 0.9 (Fig. 8).

Manual backscatter interpretation is, however, time-consuming and subjective. In later stages of the eruption, the lava flows often had lower area coverage and overlapped with previously inundated areas, resulting in minimal backscatter changes. In these areas, our estimate of the flow boundaries had greater uncertainty, and we did not produce flow boundaries for images after October 2017. Similar limitations affect coherence-based approaches, where successive emplacement events may not produce significant additional decorrelation and therefore may not be detectable. A notable advantage of backscatter-based mapping, not explored in this study, is the potential of mapping flows entering vegetated terrain (e.g., Poland 2022). Here, coherence-based mapping is also more complex due to decorrelation caused by the vegetation. Extracting the extent of the flow would require the coherence to return once the flow surface is stabilised, which would be a detectable change in signal that can be identified using a threshold or potentially a detection method like CUSUM.

The sequential analysis technique, CUSUM, shows promise for automating the flow mapping process and reducing subjectivity. Applied to SAR backscatter, CUSUM provided an objective, unbiased approach for detecting the flow emplacement. Compared to the manual-derived maps, they had lower overall agreement (MCC = 0.4, accuracy = 0.8, Fig. 8) than manual mapping. The lower agreement reflects the variability in the individual backscatter timeseries, leading to more fragmented flow detection and gaps within flows, in particular where the flow emplacement did not strongly modify surface roughness.

Developments in machine learning (ML) provide additional opportunities to improve SAR-based flow mapping. Deep learning de-speckling techniques using deep convolutional neural networks have demonstrated speckle reduction while preserving edge features (Davis et al. 2020; Valade et al. 2023). The addition of such algorithms to backscatter and potentially coherence datasets could reduce noise, aid in constraining detection limits, and potentially eliminate additional processing steps (e.g., multi-looking, boxcar filtering).

Currently, processing parameters have been developed for the 2017 Erta ‘Ale lava flows. Further work is required to evaluate their applicability to other environmental settings, SAR sensors, or varying volcanic deposits. Generally, using a smaller value for k makes CUSUM more sensitive to small shifts, therefore possibly increasing false positives and background noise, whereas a higher k value will detect larger changes, causing fewer false positives but the possibility of true changes being missed. Similarly, a smaller threshold value results in higher sensitivity and quicker detection rates, resulting in more false positive readings, whereas a larger threshold value is less sensitive and detects consistent change. For CUSUM to become a reliable monitoring tool, detection limits must be constrained to improve the accuracy of flow detection. While Erta ‘Ale’s relatively stable background provided an ideal environment for initial method development, further testing in areas with rapid surface changes related to other processes (e.g., vegetation, erosion, and weathering) is required.

#### Satellite parameters considerations

The effectiveness of mapping volcanic deposits using SAR depends strongly on sensor parameters, including wavelength, polarisation, and spatial resolution. Sensor wavelength affects both observed surface roughness and penetration depth. This affects both the observed backscatter signal and coherence preservation. Shorter wavelengths (e.g., 3.1 cm, X-band) are generally more sensitive to small-scale roughness changes and may be better suited for examining surface morphology. Whereas longer wavelengths (e.g., 23 cm, L-band) are less sensitive to subtle surface modifications and can preserve coherence over longer time intervals.

Polarisation also influences the observed backscatter signal based on the surface feature. Dual- or full-polarisation sensors (e.g., Sentinel-1, CSK-Second Generation) capture both co- and cross-polarised signals, providing different observations on surface roughness, moisture, and vegetation structure. This can be particularly useful in areas with vegetation or complex surface structures (e.g., Ferrentino et al. 2023; Solikhin et al. 2015), where single-polarisation data may be uniform. However, the increased variability introduced by polarisation can also complicate interpretation (e.g., Dualeh et al. 2021). More backscatter responses may increase the complexity of the dataset, requiring more interpretation to understand and extract flow information.

CSK imagery was used here due to its high spatial and temporal resolution over Erta ‘Ale, which was ideal as a proof-of-concept study for SAR-backscatter flow extraction. However, the increase in the number of SAR sensors now openly available offering a range of resolutions, wavelengths, and polarisation modes means that these parameters need to be considered going forward. Lower resolution sensors (e.g., Sentinel-1, 20 x 20 m, a C-band sensor (5.6 cm) with VH and VV polarisation) could provide more frequent and wide-area coverage at the cost of the spatial details. Small flows, narrow channels, or subtle morphological changes may be missed. The higher resolution of CSK allowed for the application of additional processing filters to mitigate speckle and background noise while reducing the resolution. These filters may be inappropriate on already lower resolution data as it could smooth edges and obscure fine-scale features. These satellite parameters need to be further considered when examining volcanic deposits.

## Conclusion

Spaceborne Synthetic Aperture Radar (SAR) measurements are valuable tools for remote sensing of volcanic eruptions, providing consistent observations of often dangerous and inaccessible areas. Here, we have shown that COSMO-SkyMed (CSK) SAR backscatter images can be used to measure the emplacement and behaviour of basaltic lava flows in remote areas, using the 2017 eruption of Erta ‘Ale volcano, Ethiopia, as a case study. We find that visual inspection of change detection images (ratio, difference, and RGB composites) provides the most reliable flow maps. Automated time series analysis using CUSUM, a sequential analysis technique, also produced useful flow maps, provided sufficient baseline images were available, and is less time-consuming and subjective. We observe both increases and decreases in backscatter across the flows, which correlate with local slope and can be attributed to differences in the surface roughness of flows. We conclude that backscatter can provide information on the flow surface characteristics, which, if calibrated with field observations, could be used to interpret flow morphologies. The increasing number and availability of high-resolution SAR imagery from satellites such as CSK and ICEYE means that these new techniques should be more widely applicable in the future.

**Supplementary information** Article’s accompanying supplementary information supplied as pdf file.

## Supplementary Information

Below is the link to the electronic supplementary material.

Supplementary file 1 (pdf 4585 KB)

## Data Availability

The PCA-corrected data used in analysis, along with code scripts and manually mapped flow boundaries are all freely available in a GitHub repository at: https://github.com/JemimaGosling/ErtaAle. The GitHub will be published to Zenodo following article review.
