# Supplementary material for: Analysis and automatic detection of lava flows using SAR backscatter applied to the 2017 eruption of Erta ‘Ale Volcano, Ethiopia
Source: Bull Volcanol. 2026 May 8;88(6):60. doi: 10.1007/s00445-026-01984-8 (PMC13156144; doi:10.1007/s00445-026-01984-8)
Supplement: Supplementary file 1 — (pdf 4585 KB) [file 445_2026_1984_MOESM1_ESM.pdf]

Supplementary Information for research article:

Analysis and automatic detection of lava flows using SAR backscatter applied to the 2017 eruption of Erta 'Ale Volcano, Ethiopia

Jemima Gosling[1*], Edna Warsame Dualeh[1*] and Juliet Biggs[1]

[1*]COMET, School of Earth Sciences, University of Bristol, BS8 1RJ, UK.

*Corresponding author(s). E-mail(s): xn20422@alumni.bristol.ac.uk;
edna.dualeh@bristol.ac.uk;
Contributing authors: juliet.biggs@bristol.ac.uk;

This document contains the following supplementary material:

- Visualisation of processing steps (Figure 1)
- The individual flow maps manually derived from SAR backscatter (Figure 2)
- Comparison of extracted flow parameters between manual- and coherence-derived flow maps from Moore et al. (2019) (Figure 3)

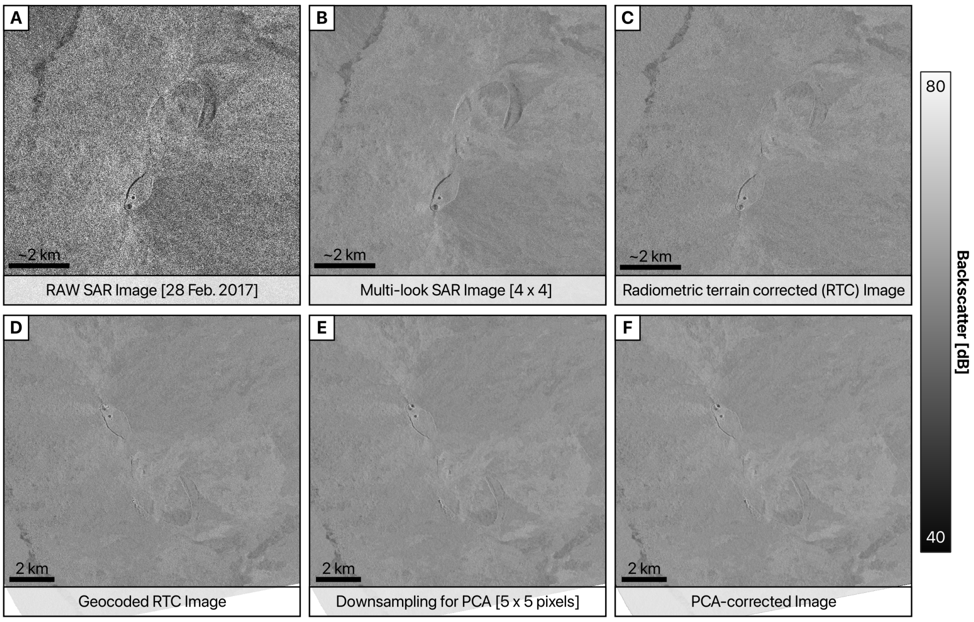

Fig. S1 COSMO-SkyMed SAR backscatter image from the 28 February 2017 showing the pre-processing steps applied. Panels show (A) the raw SAR image (full resolution), (B) 4 × 4 multi-looked image, (c) radiometric terrain correction using 30 m SRTM digital elevation model (DEM), (D) translated into geographic coordinates using the same DEM, (E) downsampling using a 5 × 5 boxcar, and (F) Principal Component Analysis (PCA) corrected image. Manual-derived maps used product (D) while the automated CUSUM processing used (F).

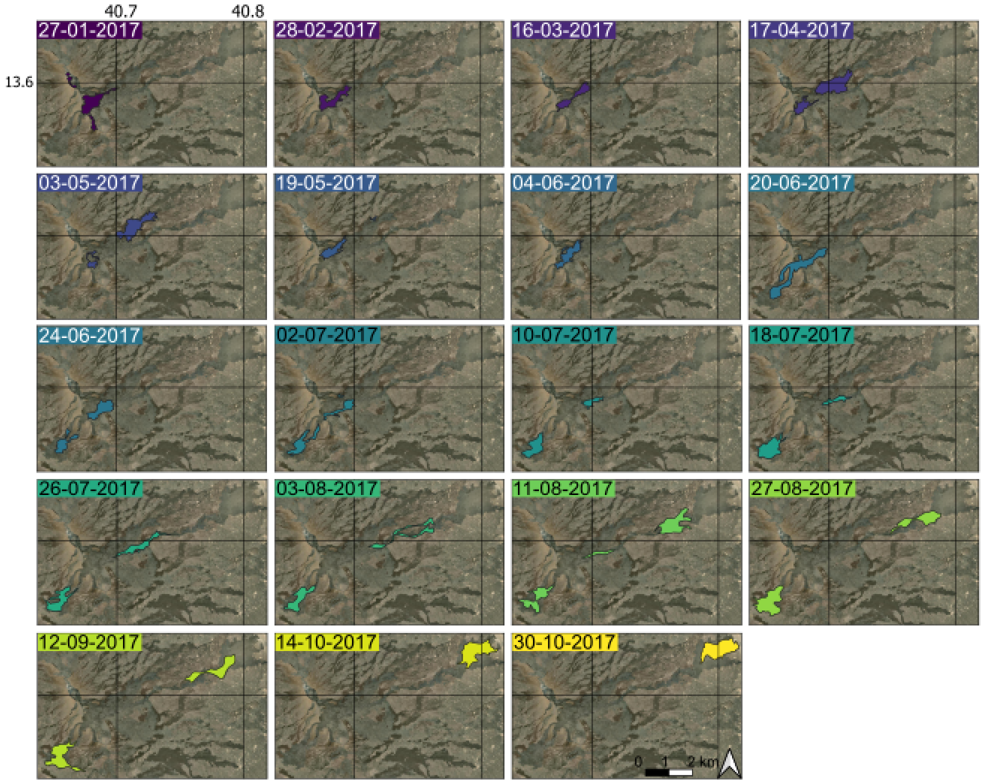

Fig. S2 Individual manual flow boundaries produced for acquisitions between January and October 2017

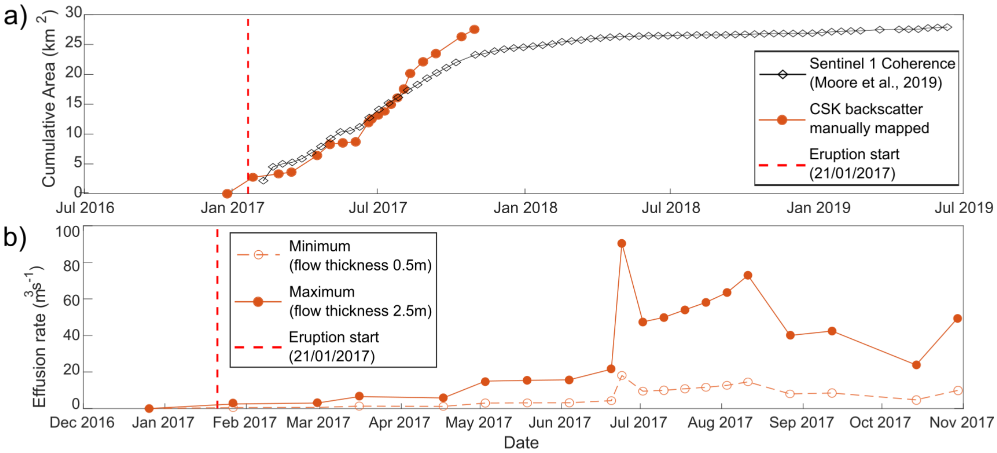

Fig. S3 (a) Manually mapped flow areas compared to Moore et al. (2019) InSAR coherence mapping and (b) Minimum and maximum effusion rates calculated from manual flow areas and flow thicknesses of 0.5 and 2.5 m respectively as stated in Moore et al. (2019)
